# Comparative Study of the Bulk and Foil Zinc Anodic Behavior Kinetics in Oxalic Acid Aqueous Solutions

**DOI:** 10.3390/ma18153635

**Published:** 2025-08-01

**Authors:** Vanya Lilova, Emil Lilov, Stephan Kozhukharov, Georgi Avdeev, Christian Girginov

**Affiliations:** 1 Department of Physics, University of Chemical Technology and Metallurgy, 8 Kliment Ohridski Blvd., 1797 Sofia, Bulgaria; emo.lilov@uctm.edu (E.L.); s.kozhukharov@uctm.edu (S.K.); 2 LAMAR–Laboratory for Advanced Materials Research, University of Chemical Technology and Metallurgy, 8 Kliment Ohridski Blvd., 1797 Sofia, Bulgaria; 3 Rostislaw Kaischew Institute of Physical Chemistry, Bulgarian Academy of Sciences, Akad. G. Bonchev Str., bl. 11, 1113 Sofia, Bulgaria; g_avdeev@ipc.bas.bg; 4 Department of Physical Chemistry, University of Chemical Technology and Metallurgy, 8 Kliment Ohridski Blvd., 1797 Sofia, Bulgaria; girginov@uctm.edu

**Keywords:** zinc anodic polarization, induction periods, temperature effects, crystallographic orientation, electrochemical behavior

## Abstract

The anodic behavior of zinc electrodes is important for energy storage, corrosion protection, electrochemical processing, and other practical applications. This study investigates the anodic galvanostatic polarization of zinc foil and bulk electrodes in aqueous oxalic acid solutions, revealing significant differences in their electrochemical behavior, particularly in induction period durations. The induction period’s duration depended on electrolyte concentration, current density, and temperature. Notably, the temperature dependence of the kinetics exhibited contrasting trends: the induction period for foil electrodes increased with temperature, while that of bulk electrodes decreased. Chemical analysis and polishing treatment comparisons showed no significant differences between the foil and bulk electrodes. However, Scanning Electron Microscopy (SEM) observations of samples anodized at different temperatures, combined with Inductively Coupled Plasma–Optical Emission Spectroscopy (ICP-OES) analysis of dissolved electrode material, provided insights into the distinct anodic behaviors. X-ray Diffraction (XRD) studies further confirmed these findings, revealing a crystallographic orientation dependence of the anodic behavior. These results provide detailed information about the electrochemical properties of zinc electrodes, with implications for optimizing their performance in various applications.

## 1. Introduction

There are several reasons to study anodic layers on zinc. The first is the wide range of practical applications these layers have. They are used for anticorrosion protection [1,2], production of pure zinc salts [3], nanoparticles with controlled shape and size [4], creating photocatalysts [5,6,7,8,9], supercapacitors [10], gas sensors [11,12], implants in surgery and biodegradable structures [13], biosensors [14], photodiodes [15], Schottky diodes [16], and other practical purposes. Another reason for studying zinc’s anodic layers is the great variety of their morphology. Anodizing zinc can produce porous layers [17,18,19], lamellar structures [20,21], cellular structures [18], needle-like arrays [20], spheres [22], arrays from nanorods [22], sponge-like and geometric structures [23], nanotubes [13,24], flower-like [25] and sunflower-like [26] structures, and others.

In the past several years, the anodic behavior of zinc has been explored in various water solutions [27,28,29,30,31]. One of the electrolytes used was an aqueous solution of oxalic acid. Interest in it arose due to the very high (over 100 volts) values of the forming voltage that were reached in galvanostatic mode. Despite the large number of published studies on the anodic behavior of zinc [32], only a few of them deal with galvanostatic anodizing [20,33,34,35,36], as does the present work. Initially, two types of electrodes, bulk and foil, were planned to be used. The bulk electrodes were intended for studying kinetics as they could be repeatedly used. However, the bulk electrodes are not suitable for studying the composition and morphology of the resulting films using techniques like XRD, SEM, AFM, etc. Therefore, electrodes cut from zinc foil were used instead. It was found that the anodic behavior of the two types of electrodes differed significantly. This difference is not described in the review articles [27,37,38,39,40,41,42,43]. Commonly, researchers used either bulk electrodes [34] or those cut from foils [44]. That is why there is no data regarding the difference between these types of electrodes. This fact has inspired the present study. Particularly, the present work is focused on two purposes: (i) to describe the differences in the kinetics of the two types of electrodes, and (ii) to identify the reasons for these differences.

## 2. Materials and Methods

### 2.1. Sample Preparation and Kinetic Studies

Two types of electrodes were used in the experiments:

(i) Bulk electrodes: Pure zinc (p.a.) was melted in a sealed, evacuated tube of fused silica at 700 °C. It was then quenched in water with ice at about 0 °C to obtain a zinc rod. Cylindrical pieces were cut from this rod, and one of the bases was used as a working surface with an area of 0.8 cm^2^. A thick Cu wire was soldered to the other base. All surfaces except the working one were isolated with a thick layer of epoxy resin. The bare surface was treated with emery paper and then polished with a polishing paste with a grain size down to 0.5 µm. After this, the electrode was washed with neutral detergent and double-distilled water.
(ii) Foil electrodes: The second type of electrode, with an area of 2 cm^2^, was cut out of zinc foil (Alfa Aesar 99.98%). They were degreased with nital and washed with distilled water. The working surfaces were electropolished in an alcoholic solution of phosphoric acid for 20 min at an electric potential of 20 V and 5 °C. The electrodes were then washed with double-distilled water.

Aqueous solutions of oxalic acid with concentrations varied between 0.005 mol dm^−3^ and 0.5 mol dm^−3^ were used as forming electrolytes. The current densities were varied from 1mA cm^−2^. to 40 mA cm^−2^.

The anodization process was carried out in a two-electrode cell with a gold cathode at 293 K, except for studying the dependence of the anodic behavior on temperature.

### 2.2. Characterization Methods

The Inductively Coupled Plasma–Optical Emission Spectroscopy (ICP-OES) study was performed with a Prodigy High Dispersion ICP-OES, Teledyne Leeman Labs, (Hudson, NH, USA). The concentration of zinc was determined at a wavelength of 213 nm.

The Scanning Electron Microscopy (SEM) study was performed with a Tescan SEM/FIB LYRA I XMU device, Tescan Group, (Brno-Kohoutovice, Czech Republic) in the regime of secondary electrons.

X-ray diffraction patterns were recorded in an angle interval from 7° to 90° (2θ) by a Philips PW 1050 (Almelo, Nederland) diffractometer equipped with CuK_α_ tube and a scintillation detector.

The problem of result reproducibility is of great importance in research concerning the anodic behavior of valve metals. This is even more important when dealing with induction periods, where the standard deviation can exceed 40% in these studies. Therefore, in cases where necessary, seven measurements were conducted to establish the mean value and calculate the standard deviation.

## 3. Results and Discussion

**Figure 1:**
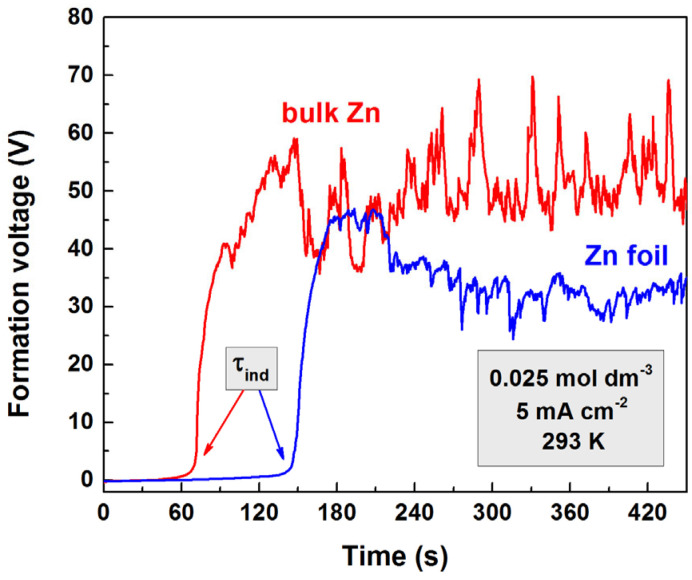
Kinetic curves for bulk zinc and foil electrodes polarized anodically in 0.025 mol dm^−3^ solution of oxalic acid with a current density of 5 mA cm^−2^.

Typical kinetic curves are shown in Figure 1. There is no significant change in the electric potential at the beginning of the process, which is the induction period (denoted as τ_ind_ in Figure 1). After this period, a rapid increase in the forming voltage is observed. The further increase in voltage is limited by breakdowns. The parameters of the kinetic curve (induction period duration, slope of the voltage increase, and breakdown voltage) depend on the conditions of the anodic polarization (forming electrolyte concentration, current density, and temperature) [34,35]. No clear dependence was found for the breakdown voltage and slope on the concentration of the forming electrolyte and current density, nor on temperature. Clear relations were only found for the induction period durations, which is the focus of the present work.

### 3.1. Dependence of Induction Period Durations on the Forming Electrolyte Concentration

**Figure 2:**
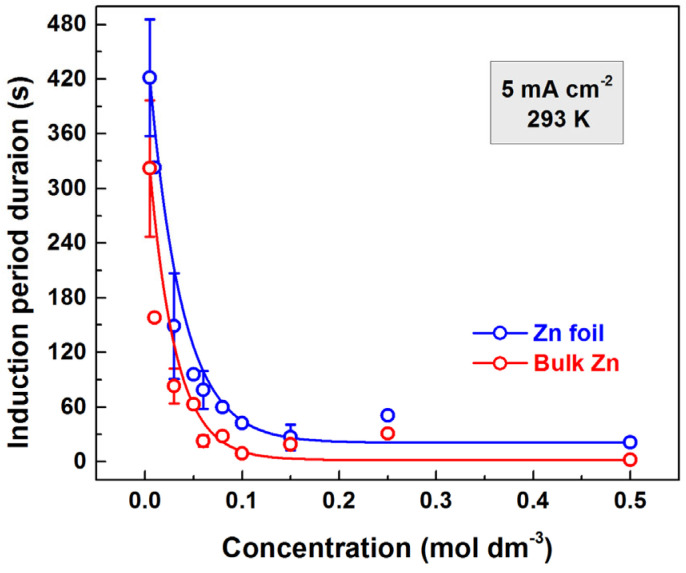
Dependence of the induction period on the concentration of the forming electrolyte for bulk and foil zinc anodized with a current density of 5 mA cm^−2^.

The anodic polarization of valve metals is commonly carried out in aggressive media, such as acids and bases. These media usually become more aggressive as the concentration increases. As a result, the process of film dissolution, which slows down the film formation process, intensifies with an increasing forming electrolyte concentration. In the presence of induction periods, their durations increase with the increasing solution concentration. However, the anodization of zinc has shown the exact opposite trend. Hence, the induction period durations decrease with increasing concentration, as shown in Figure 2. For the curves shown there, the current density of 5 mA cm^−2^ was selected so that the induction period duration would be relatively long, minimizing experimental error. At the same time, the voltage increase at the end of the induction period needed to be sufficiently rapid so that voltage fluctuations would not interfere with determining its endpoint.

**Figure 3:**
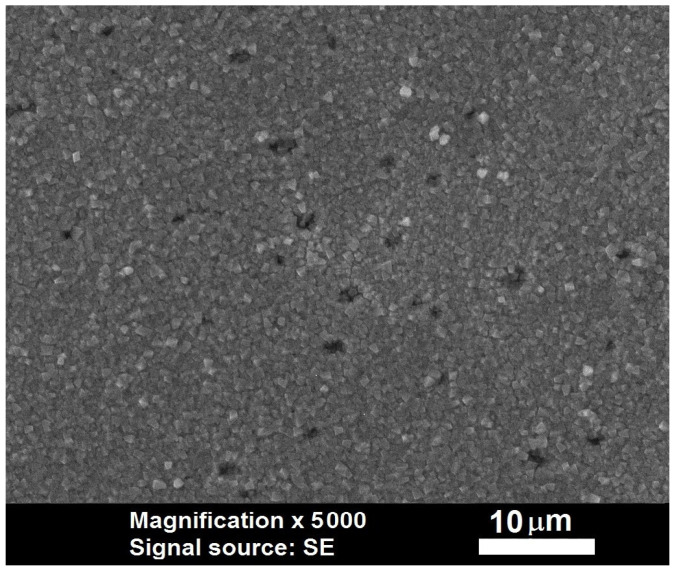
SEM micrographs of foil zinc anodized for 50 s in 0.06 mol dm^−3^ solution of oxalic acid with a current density of 5 mA cm^−2^ stopped at the very end of the induction period.

This behavior occurs because the anodic film consists of zinc oxalate crystals [35]. At the beginning of the anodic polarization, these crystals are formed on the electrode surface but do not completely cover the entire area. There are spaces between them, even at the end of the induction period, as seen in Figure 3.

The metal is in direct contact with the solution, and the potential is low. As the anodic polarization continues, the size of the crystals grows. When they cover the entire surface, the induction period is concluded. The more concentrated the electrolyte, the faster the dimensions of the crystals increase and the shorter the induction period [35].

There is a noticeable difference between the two types of electrodes. The induction periods are much longer for the foil samples, as can be seen from Figure 2. Their durations decrease as solution concentration increases, but never reach zero.

### 3.2. Dependence of Induction Period Durations on the Applied Current Density

**Figure 4:**
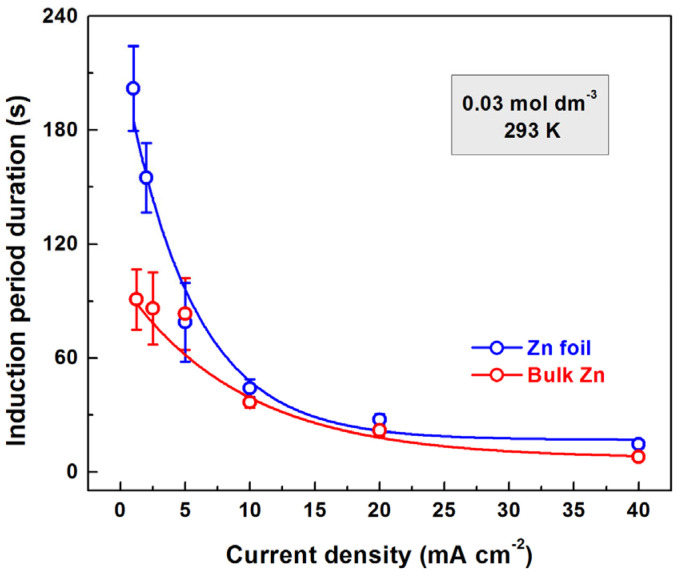
Dependence of the induction period duration on the current density for bulk and foil zinc anodized in 0.03 mol dm^−3^ oxalic acid.

The dependence of the induction period on current density for zinc anodization in a 0.03 mol dm^−3^ solution of oxalic acid is illustrated in Figure 4. A second-degree curve approximation indicates that this dependence never intercepts the *X*-axis.

### 3.3. Dependence of Induction Period Durations on the Forming Electrolyte Temperature

The most noteworthy result of this study was obtained while investigating the dependence of the induction period duration on temperature. The investigation was carried out between 278 and 333 K. This temperature range was selected, because at lower temperatures (around the freezing point), changes in electrolyte properties are expected [45], whereas temperatures above 333 K cause intense evaporation of the solution. Particularly, at temperatures approaching the freezing point, the electrolyte viscosity is rather high, suppressing the ionic mobility [45].

**Figure 5:**
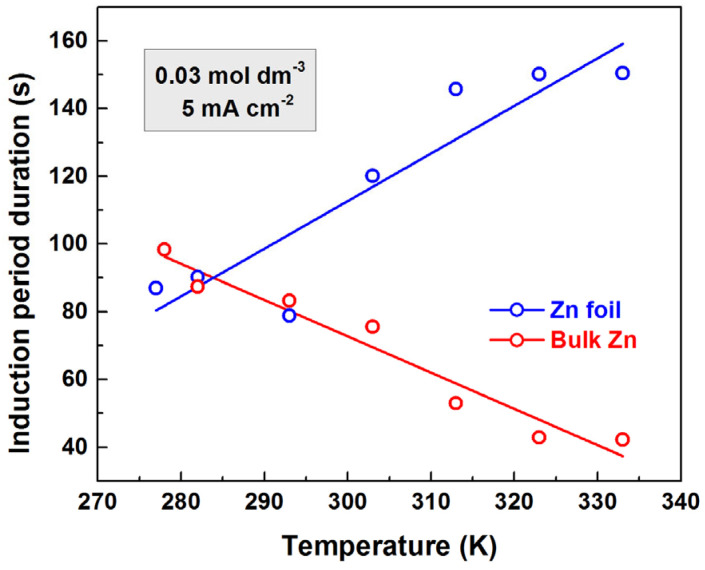
Dependence of the induction period durations on the temperature for bulk and foil electrodes treated in 0.03 mol dm^−3^ aqueous solution of oxalic acid with current density 5 mA cm^−2^.

Two opposing processes take place in the case of valve metal anodic polarization. The former process is the formation of a layer on the electrode surface, while the latter is the dissolution of this film. The process of dissolution becomes more intensive as the temperature increases. As a result, the induction period duration is expected to increase with the increase in temperature. This expectation was observed in the case of anodic polarization of foil, as shown in Figure 5. However, the results for bulk zinc anodic polarization showed an opposite trend. At low solution temperatures (around 283 K), the induction period durations were almost equal (about 90 s) for both foil and bulk electrodes. At high electrolyte temperatures, the induction period durations decreased more than twice in the case of bulk electrodes while increasing about twice in the case of foil electrodes. The error bars were removed from the figures to enhance the comparability of the dependencies. The standard deviation for the relationships depicted in Figure 5 is less than 38%.

The effect of temperature on the formation of anodic films on zinc is dependent on the type of electrolyte used. The anodic behavior of zinc in aqueous solutions of potassium, sodium, and ammonium bicarbonate was studied [46], and it was found that an increase in the temperature of just 15 °C led to a fivefold increase in the growth rate for NaHCO_3_ and NH_4_HCO_3_. However, for zinc anodes in alkaline electrolytes, an increase in temperature leads to a rise in passivation time [47]. In aqueous borate electrolytes, the duration of the induction period also increases with temperature during zinc anodizing [34].

Several possible reasons may account for the disparities between bulk and foil electrodes. One potential explanation could be variations in the chemical compositions of the bulk and the foil electrodes. To verify whether the compositions of the electrodes are distinguishable, chemical analyses of both electrodes were conducted. The ICP-OES study of the chemical composition revealed only insignificant differences between the bulk and foil electrode compositions. Thus, the only notable difference was the Ca content, which was 0.014% for the foil and 0.019% for the bulk material. In addition, for the other elements, the difference was an entire order of magnitude lower. Hence, these differences are not sufficient to account for the disparities.

An experiment was conducted to determine whether the varying kinetics were a result of different polishing treatments. To this end, the induction periods of mechanically polished and electropolished electrodes of both types were compared at a temperature of 323 K, where the difference in induction periods between the two types of electrodes was almost four times. The induction period (τ_ind_) for mechanically polished bulk samples was (42.9 ± 13.5) s, whereas for electropolished samples, it was (50.6 ± 19.2) s. The τ_ind_ for mechanically polished foil was (130.0 ± 50.8) s, and for electropolished foil, it was (150.2 ± 65.7) s. These results suggest that the temperature dependence of the induction period duration is not a result of the sample preparation method.

**Figure 6:**
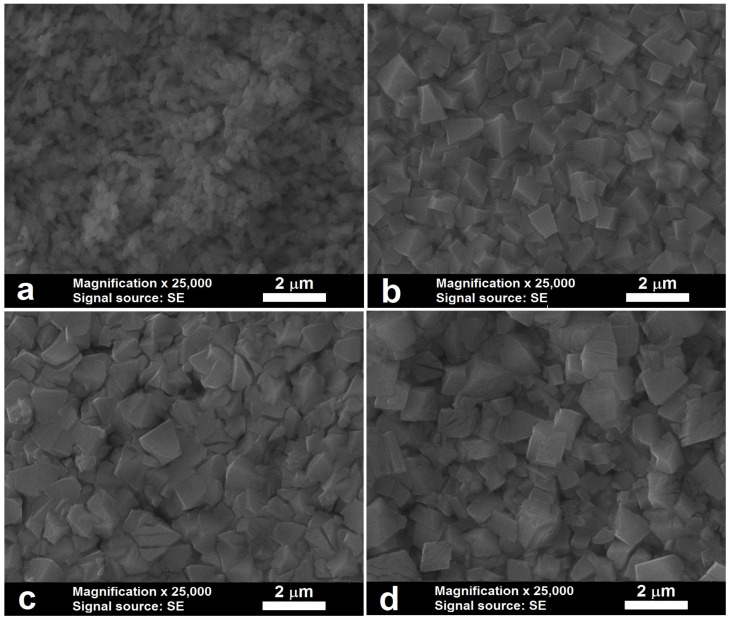
SEM images of zinc foil samples anodized at (**a**) 278 K and (**b**) 333 K and bulk samples anodized at (**c**) 278 K and (**d**) 333 K. The samples were anodized in a 0.03 mol dm^−3^ aqueous oxalic acid solution with a current density of 3.85 mA cm^−2^ for 40 s.

To determine the cause of the varying anodic behavior between bulk zinc electrodes and those cut from foil, an electron microscopy study was conducted. The results of this study are depicted in Figure 6 through a series of images.

The images reveal that at a lower temperature, no crystals are present on the foil surface (Figure 6a), but at a higher temperature, crystal formation is evident (Figure 6b). For the bulk samples at a lower temperature, the crystals are uniform in size (Figure 6c). However, at a higher temperature, smaller crystals are observed, and the larger ones show signs of secondary dissolution (Figure 6d). The images provide insight into the temperature dependence of the induction period duration. Based on the images, the following explanation can be offered. According to some studies [12,25,48,49], zinc anodization starts with the electrochemical dissolution of the electrode material, followed by the deposition of the dissolved products on the substrate surface as nanoparticles of varying morphologies. The zinc foil dissolves more readily compared to the bulk samples, likely due to differences in the crystallographic planes exposed to dissolution [50,51,52]. At low temperatures, the solubility of the reaction products is low, leading to rapid coverage of the electrode surface with compounds that do not have time to crystallize (Figure 6a), resulting in a short induction period. At higher temperatures, the solubility of the electrochemical dissolution products increases, leading to slower saturation of the electrolyte around the electrode surface and sufficient time for the precipitated products to crystallize (Figure 6b), resulting in a longer induction period.

For the bulk samples, the weaker dissolution leads to slower saturation of the electrolyte around the electrode surface, allowing for sufficient time for the products to crystallize. As a result, the induction periods at low temperatures are longer compared to those for the foil. With increasing temperature, the chemical dissolution of the formed crystals also increases, leading to secondary dissolution of the larger crystals (observed in Figure 6d), which enables the formation of new, smaller crystals. The electrode surface is covered more rapidly, reducing the induction period duration.

**Figure 7:**
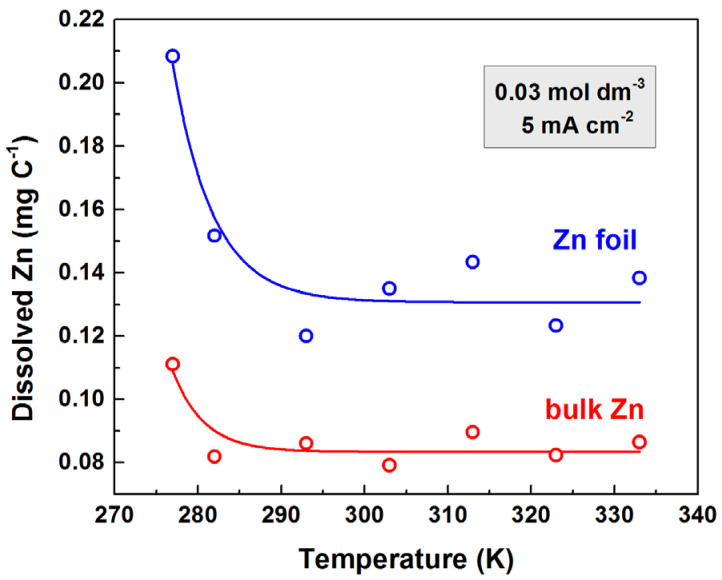
Dependence of the dissolved zinc on the temperature for bulk and foil electrodes treated anodically in 0.03 mol dm^−3^ oxalic acid with a current density of 5 mA cm^−2^.

The hypothesis that the foil dissolves more intensively than the bulk material is supported by the temperature-dependent dissolution shown in Figure 7. The data show that the foil dissolves approximately twice as much as the bulk material.

**Figure 8:**
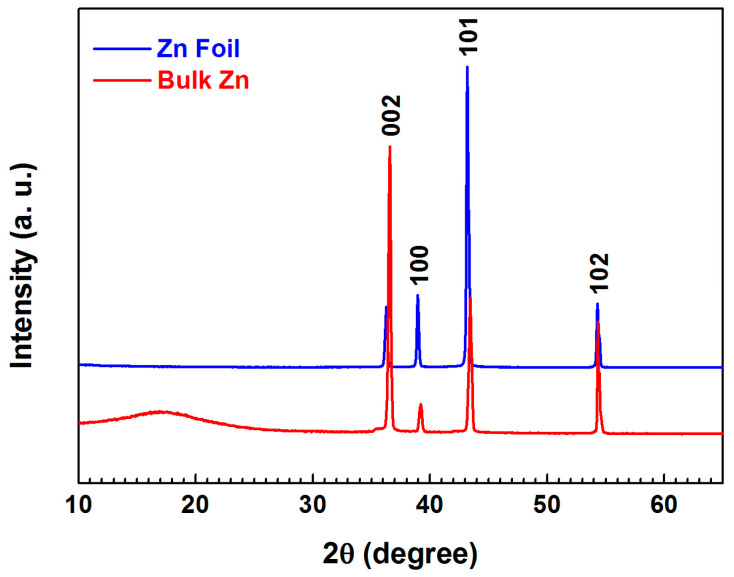
XRD patterns for foil and bulk electrodes.

The X-ray diffractograms of non-anodized samples from both electrode types confirm that the different kinetics between the bulk electrode and the one cut from the foil can be attributed to a distinct orientation of the crystallites composing them (Figure 8).

From the figure, it can be observed that the crystallites constituting the zinc foil are predominantly oriented with their (101) crystallographic plane relative to the working surface. On the other hand, the crystallites forming the bulk electrode are oriented with their (002) plane. Crystalline materials are inherently anisotropic, which means that their properties vary in different directions. Previous studies [51] have shown that the (002) and (101) planes have a higher passivation potential compared to the (112) plane. Furthermore, layers of zinc ions deposit more easily on the (112) surface than on the (002) surface [52]. As a result, the induction periods for the bulk electrodes are shorter and further decrease with increasing temperature, while for the foil, these are longer and increase with temperature.

## 4. Conclusions

The kinetics of the anodic behavior of bulk and foil zinc electrodes were compared. It was found that the duration of the induction periods depends on the concentration of the forming electrolyte, the current density, and the temperature at which the process is carried out. It decreases with increasing electrolyte concentration and current density, being significantly shorter for bulk electrodes than for foil ones.

The most notable difference was observed when examining the correlation between the duration of the induction period and temperature. Specifically, for bulk electrodes, the duration of the induction period decreases, whereas for foil electrodes it increases as the temperature increases.

To determine the reasons for these differences, the chemical composition of the two types of electrodes was compared by ICP-OES. Although a slight variation in calcium content was identified, it was insufficient to explain the disparities in anodic behavior.

To eliminate the possibility that differences in sample preparation could account for the observed differences in kinetics, an experiment was conducted, and the results indicated that this was not the case either.

An explanation of the observed difference in kinetics can be made based on the SEM studies of the obtained layers. According to it, initially, the surface of the metal is dissolved electrochemically, and subsequently, the products of this dissolution cover the electrode, forming nanoparticles of different shapes and sizes. The zinc foil dissolves faster due to the difference in the crystallographic surfaces subjected to dissolution. At low temperatures, the solution around the surface saturates quickly, resulting in shorter induction periods. At high temperatures, the solution saturates more slowly, and its duration increases.

For bulk electrodes, dissolution is weaker, and its products crystallize on the surface. As the temperature increases, secondary dissolution and formation of new crystals occur, resulting in a shortening of the duration of the induction period.

This explanation is confirmed by the examination of the temperature dependence of the amount of dissolved electrode material, which shows that the foil dissolves about twice as much as the bulk material.

Furthermore, the obtained XRD patterns of the non-anodized electrodes reveal that the bulk electrodes and those cut from the foil predominantly exhibit crystallites oriented with different crystallographic surfaces towards the electrode’s working surface.

These observations could be useful to fellow researchers working in this field. It would be of great interest to verify whether the structures formed on the surface of the zinc electrodes during anodic polarization also depend on the orientation of the crystallites.

## Data Availability

The data presented in this study are available on request from the corresponding author. The data are not publicly available due to privacy concerns.
